# Antioxidant Activity and Phytochemical Profiling of Steam-Distilled Oil of Flaxseed (*Linum usitatissimum*): Therapeutic Targeting Against Glaucoma, Oxidative Stress, Cholinergic Imbalance, and Diabetes

**DOI:** 10.3390/molecules30163384

**Published:** 2025-08-14

**Authors:** İlhami Gulcin, Muzaffer Mutlu, Zeynebe Bingol, Eda Mehtap Ozden, Ziba Mirzaee, Ahmet C. Goren, Ekrem Köksal

**Affiliations:** 1 Department of Chemistry, Faculty of Science, Ataturk University, Erzurum 25240, Türkiye; edamehtap3@gmail.com; 2 Rectorate of Agri İbrahim Çeçen University, Agri 04100, Türkiye; 3 Vocational School of Applied Sciences, Gelişim University, Istanbul 34315, Türkiye; muzaffermutlu@hotmail.com; 4 Department of Medical Services and Techniques, Tokat Vocational School of Health Services, Gaziosmanpasa University, Tokat 60250, Türkiye; zeynep.bingol196@gmail.com; 5 Department Chemistry, Faculty of Sciences, Gebze Technical University, Kocaeli 41400, Türkiye; acgoren@gtu.edu.tr; 6 Troyasil HPLC Column Technologies, Doruk Analitik, Mehmet Akif Mah. Yumurcak Sok. No:43, Ümraniye, Istanbul 34774, Türkiye; 7 Department of Chemistry, Faculty of Science and Arts, Erzincan Binali Yildirim University, Erzincan 24100, Türkiye; koksalekrem@gmail.com

**Keywords:** *Linum usitatissimum*, radical scavenging, metabolic disorders, GC/MS, LC-HRMS

## Abstract

This investigation explored the chemical constituents and biological activities of the steam-distilled oil of *L. usitatissimum* (SDOLU), employing sophisticated techniques including LC-HRMS, GC-MS, and GC-FID. The analysis identified a diverse array of 17 phenolic compounds, with linoleoyl chloride (64.05%) and linoleic acid (10.39%) as the major fatty acid components. The SDOLU demonstrated remarkable antioxidant capacity, effectively neutralizing free radicals in both DPPH^•^ (IC_50_: 19.80 μg/mL) and ABTS^•+^ (IC_50_: 57.75 μg/mL) scavenging assays, alongside robust electron-donating activity in reducing ability tests. Moreover, the SDOLU showed significant inhibition of key enzymes implicated in metabolic and neurodegenerative disorders, including α-amylase (IC_50_: 531.44 μg/mL), acetylcholinesterase (IC_50_: 13.23 μg/mL), and carbonic anhydrase II (IC_50_: 281.02 μg/mL). Collectively, these results highlight the SDOLU as a valuable natural source of multifunctional bioactivities with potential applications in combating oxidative stress and enzyme-related global diseases. Further studies are warranted to validate its therapeutic efficacy and expand its industrial utilization.

## 1. Introduction

Vegetable oil is a vital part of the daily human diet, valued not only for enhancing flavor and texture in cooking but also for providing a key source of energy that helps regulate and maintain normal body temperature [1]. Vegetable oil provides essential fatty acids that aid in vitamin absorption and hormone and prostaglandin production. Also, they help support immunity and prevent chronic diseases [2]. Flaxseed oil is rich in unsaturated fatty acids, phenolics, and phytosterols, which contribute to its antioxidant, anti-inflammatory, antidiabetic, and cardiovascular protective effects. It supports blood sugar control and may help prevent chronic diseases. However, like many minor vegetable oils, more research is needed to fully understand its long-term health benefits [3]. Flaxseed is one of the best plant-based sources of ω-3 fatty acids [4]. Flaxseed contains two major bioactive components: α-linolenic acid, a polyunsaturated ω-3 fatty acid that constitutes about 59% of its oil content, and secoisolariciresinol diglucoside, a lignan present at approximately 0.7% to 1.9% of the whole seed by weight [5]. Flaxseed oil also contains a diverse range of bioactive compounds, such as linoleic acid (an ω-6 fatty acid), plant lignans, cyclic peptides, complex polysaccharides, various alkaloids, naturally occurring cyanogenic glycosides, and trace levels of cadmium [6].

Reactive oxygen species (ROS) are highly reactive molecules and ions derived from oxygen, including free radicals such as superoxide (O_2_·−) and hydroxyl (OH·), as well as nonradical species like hydrogen peroxide (H_2_O_2_) and ozone (O_3_) [7]. While ROS are naturally produced during normal metabolism and play critical roles in cell signaling, gene regulation, and immune defense, their overproduction can lead to oxidative damage associated with aging and various diseases, including cancer, cardiovascular, and neurodegenerative disorders [8,9]. To counteract these harmful effects, antioxidant systems—such as superoxide dismutase and glutathione—maintain redox balance and protect cells from oxidative stress [7]. Antioxidants are also vital in food systems, where they prevent lipid and protein oxidation, preserving flavor, color, and texture during storage [10,11]. Dietary antioxidants, primarily phenolics and flavonoids abundant in fruits and vegetables, contribute to reduced risks of chronic diseases by protecting biomolecules from oxidative damage [12]. Their effectiveness depends on chemical structure and bioavailability, with recent research highlighting compounds like bromophenols for their antioxidant and enzyme inhibition properties relevant to disease prevention [13,14]. Overall, antioxidants play a crucial role in maintaining both food quality and human health by mitigating oxidative stress and cellular damage [15].

Alzheimer’s disease (AD) is the leading cause of dementia and is rapidly emerging as one of the costliest, deadly, and burdensome diseases of the 21st century. Recent advances have deepened our understanding of AD, from its genetics and preclinical stages to improved early diagnosis through biomarkers and imaging. These developments are paving the way for earlier, more effective, and combined treatment strategies [16]. Currently, around 55 million people globally have AD, and this number is expected to double every 5 years [17]. In developed countries, about one in 10 adults aged 65 and over show early signs of AD, while over one-third of those aged 85 and older may experience advanced symptoms [18]. Population studies in Europe show that AD prevalence rises sharply with age—from 0.6% in those aged 65–69 to 22.2% in individuals aged 90 and above—confirming global trends in the disease’s increasing prevalence [19,20].

Diabetes mellitus (DM) is a widespread metabolic disorder affecting over 350 million people globally and is a leading cause of morbidity and mortality. The two main types are Type-1 diabetes mellitus (T1DM), caused by autoimmune destruction of pancreatic beta cells, and Type-2 diabetes mellitus (T2DM), the more common form resulting from insulin resistance in tissues like the liver and muscles [21]. Both types are associated with serious microvascular complications such as diabetic nephropathy, retinopathy, and cardiomyopathy [22]. Diabetic nephropathy affects 20–30% of T2DM patients and up to 30% of those with T1DM. About one-third of diabetics develop diabetic retinopathy, with over 10% facing vision-threatening conditions [23,24]. With over 450 million people affected worldwide in 2017 and projections reaching 629 million by 2045, T2DM represents a major global health challenge that demands new management approaches [25].

Glaucoma is a group of optic neuropathies marked by progressive loss of retinal ganglion cells, leading to optic nerve damage and irreversible vision loss [26]. It affects over 90 million people globally, making it the leading cause of irreversible blindness [27]. The disease often remains symptom-free until advanced stages, resulting in many undiagnosed cases, especially in low- and middle-income countries, where over 90% go undetected [28]. Glaucoma is mainly classified into open-angle and angle-closure types; open-angle glaucoma accounts for over 80% of cases in the United States, but angle-closure glaucoma causes more severe vision loss [29,30]. Secondary glaucoma may result from trauma, corticosteroid use, inflammation, tumors, or other ocular conditions. Although lost vision cannot be restored, early detection and treatment can slow progression and preserve sight [31]. With aging populations, glaucoma prevalence is expected to rise significantly, underscoring the need for improved screening and care to prevent avoidable blindness [32].

This study employs advanced analytical techniques such as LC-HRMS and GC-MS/FID to investigate the complex chemical profile of volatile constituents of *L. usitatissimum*, focusing on its potential to combat oxidative stress, a key factor in diseases like glaucoma and diabetes. By identifying bioactive compounds including polyphenols and volatiles, fatty acids and steroids substances, the research explores how the oil may help restore cholinergic balance and reduce oxidative damage associated with neurodegenerative conditions such as AD. Additionally, this work addresses important gaps in understanding the role of fatty acids of the seeds of this species in metabolic and ocular health. The promising antioxidant and enzyme-modulating properties revealed highlight the need for further research into the oil’s therapeutic applications in managing glaucoma, diabetes, and related oxidative stress disorders.

## 2. Results

### 2.1. Polyphenol Profile of Volatile Constituents of L. usitatissimum

The LC-HRMS assay validation covered essential parameters, including linearity, precision, selectivity, accuracy, matrix effects, recovery, and analyte stability [33,34]. The major compounds detected were Epigallocatechin (1.94 mg/L) and Naringenin (1.22 mg/L). To analyze these secondary metabolites, the oil was prepared via liquid-liquid extraction.

**Figure 1:**
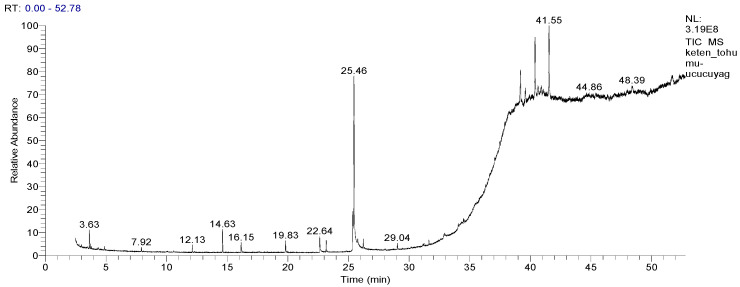
Using GC-MS, various compounds in steam-distilled oil of *L. usitatissimum* (SDOLU) were identified and quantified by percentage.

The composition of the oil was also analyzed in detail using GC-MS technique [35]. Considering the GC-MS data, the presence of six major components in the oil was determined, including linolenic acid (57.97%), linoleic acid (13.21%), and palmitic acid (4.91 %). Other significant compounds were determined as steroidal compounds such as campesterol (2.49 %), stigmasterol (0.4 %), sitosterol (5.2%), and cycloartenol (6.56 %). These findings, presented in Figure 1 and Table 1, reveal the rich profile of the steam-distilled oil of *L. usitatissimum* (SDOLU).

Recently, there have been intensive studies on the extraction methods for flaxseed oil and its content variability. Its flavor profile is generally and primarily composed of small-molecule alcohols, aldehydes, ketones, esters, pyrazines, furans, and pyrroles. These compounds are formed mainly through lipid oxidation, Maillard reactions, and the breakdown of branched-chain amino acids [36]. From a processing standpoint, flaxseed oil can generally be divided into cold-pressed and hot-pressed varieties [37]. Yang et al. carried out comprehensive studies to differentiate the flavor profiles of these two types [38]. Cold-pressed flaxseed oil typically retains a natural, mild aroma, while hot-pressed flaxseed oil develops a pronounced roasted scent. According to Han et al., aldehydes are the dominant contributors to the overall aroma of flaxseed oil [39]. In contrast, although alkanes give off a plant-like scent, they are chemically unstable and have high odor thresholds, thus playing a minor role in the overall flavor. Alcohols and esters are present in lower concentrations and, due to their relatively high odor thresholds, contribute minimally to flaxseed oil’s flavor as well. To study how temperature affects flaxseed oil’s aroma profile, Sun et al. applied headspace gas chromatography–ion mobility spectrometry combined with principal component analysis [36]. Meanwhile, Ma et al. employed conventional gas chromatography–mass spectrometry (GC–MS) to evaluate the volatile compounds generated during various roasting periods [40]. Their analysis identified 51 compounds across 11 aroma categories and mapped their trends over the course of heating. In one of these studies, the lipid profile of flaxseed oil, particularly in relation to flavor generation in hot-pressed oils, was reported [41]. This study aimed to explore the potential lipid precursors of flaxseed oil by examining the relationships between specific lipid markers and major aroma-active compounds. In total, 94 volatile compounds were detected using headspace solid-phase microextraction gas chromatography–mass spectrometry and sniffing technology. These flavor compounds increased from 3483.8 μg/Kg (0 min) to 83,814.5 μg/Kg (30 min). Nine odor-active compounds were selected based on odor activity value. Furthermore, 358 lipid molecules were identified using ultra-performance liquid chromatography coupled with quadrupole time-of-flight mass spectrometry, of which 139 were selected as lipid markers. Correlation analysis revealed significant correlations between the nine flavor markers and the levels of glycerophosphatides and glycerol lipids. Notably, 2-methylpropanal, 2-methylbutanal, and 2,5-dimethylpyrazine exhibited the same trends, consistent with precursors in the lipid degradation pathway. These results help elucidate the metabolic pathways involved in flaxseed oil flavor reported [41].

While it is well known that the flavor profile of flaxseed oil is influenced by thermal processing, the precise contribution of lipids to flavor formation is not yet fully clarified. Lipids represent a complex class of biomolecules with various nutritional and functional bioactivities [42], and they play a crucial role as precursors in the formation of low-molecular-weight flavor compounds [43]. Seed lipids are classified into eight main categories: fatty acyls, glycerolipids, glycerophospholipids, prenol lipids, sphingolipids, sterol lipids, and saccharolipids [44]. These lipids enhance the complexity of food flavor either through their degradation into volatile compounds or via interactions with other substances during processes like the Maillard reaction, Strecker degradation, and various stages of food processing, cooking, and storage [45].

In addition, Liao et al. utilized ultra-performance liquid chromatography combined with quadrupole time-of-flight mass spectrometry (UPLC-Q-TOF-MS) to analyze the lipid composition of flaxseed oil, identifying 668 lipid molecules spanning 15 distinct lipid classes [46]. As lipid degradation is a key metabolic route in the formation of volatile aroma compounds, this has led to growing interest in exploring the relationship between lipidomic and flavoromic profiles. UPLC-Q-TOF-MS offers high sensitivity, superior resolution, and precise qualitative capabilities for both targeted and untargeted lipidomic analyses. In recent years, it has been increasingly utilized in comprehensive food lipidomics studies [47].

### 2.2. Determination of Reducing Power in Steam-Distilled Oil of L. usitatissimum

**Figure 2:**
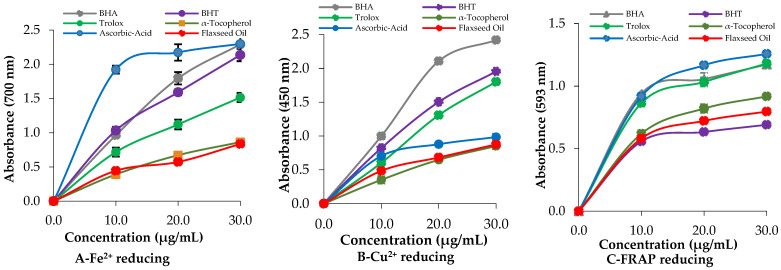
Reduction potentials of Fe^3+^ (**A**), Cu^2+^ (**B**), and Fe^3+^-TPTZ (**C**) by steam-distilled oil of *L. usitatissimum* (SDOLU) and standards.

*L. usitatissimum* oil demonstrated notable reducing power in assays involving Fe[Fe(CN)_6_]^3^−, Fe^3+^-TPTZ, and Cu^2+^ reducing abilities [48]. To evaluate its reduction potential, a conversion assay was conducted to measure the Fe^3+^ to Fe^2+^ interconversion (Figure 2A and Table 2). At the concentration of 50 µg/mL, both the SDOLU and the standards exhibited Fe^3+^-reducing activity (*p* < 0.01), with a strong overall correlation (r^2^ = 0.9804). The reducing capacities, ranked from highest to lowest based on absorbance values, were as follows: Ascorbic acid (2.298 ± 0.086, r^2^ = 0.9659) ≥ BHA (2.292 ± 0.012, r^2^ = 0.9993) ≥ BHT (2.136 ± 0.090, r^2^ = 0.9957) > Trolox (1.514 ± 0.066, r^2^ = 0.9963) > SDOLU (1.005 ± 0.043, r^2^ = 0.9997) > α-Tocopherol (0.862 ± 0.038, r^2^ = 0.9996).

The increase in absorbance indicates the formation of a colored complex, signifying a higher reducing capacity (Figure 2A). Moreover, the Fe^3+^-TPTZ and Cu^2+^-reducing abilities of the SDOLU were also evaluated, with results detailed in Figure 2B,C, and Table 2. The tested oil exhibited strong absorbance values across the tested concentrations, indicating notable reducing activity. At the concentration of 30 μg/mL, the SDOLU and the standards reduced Cu^2+^ ions (Figure 2B). The reducing capacities, ranked from highest to lowest based on absorbance values, were as follows: BHA (2.418 ± 0.018, r^2^ = 0.9887) > BHT (1.953 ± 0.045, r^2^ = 0.9998) > Trolox (1.800 ± 0.096, r^2^ = 0.9974) > Ascorbic acid (0.983 ± 0.048, r^2^ = 0.9822) > SDOLU (0.875 ± 0.028, r^2^ = 0.9907) > α-Tocopherol (0.851 ± 0.046, r^2^ = 0.9994).

Although BHA showed the highest absorbance, the oil demonstrated a relatively strong Cu^2+^ reducing potential compared to most natural standards. The reducing potential of the SDOLU was evaluated using the FRAP assay, with the results summarized in Table 3 and Figure 2C. The tested oil demonstrated significant FRAP reducing capacity, reflecting effective antioxidant activity relative to standard compounds. The absorbance values were ranked from highest to lowest as follows: Ascorbic acid (1.257 ± 0.024, r^2^ = 0.9869) > Trolox (1.180 ± 0.032, r^2^ = 0.9732) > BHA (1.172 ± 0.014, r^2^ = 0.9605) > α-Tocopherol (0.918 ± 0.011, r^2^ = 0.9904) > SDOLU (0.796 ± 0.010, r^2^ = 0.9821) > BHT (0.690 ± 0.008, r^2^ = 0.9645). SDOLU exhibited a higher reducing capacity than BHT and approached that of α-tocopherol, indicating its potential as a natural antioxidant with considerable efficacy in combating oxidative stress.

### 2.3. Radical Scavenging Activity of the Steam-Distilled Oil of L. usitatissimum

**Figure 3:**
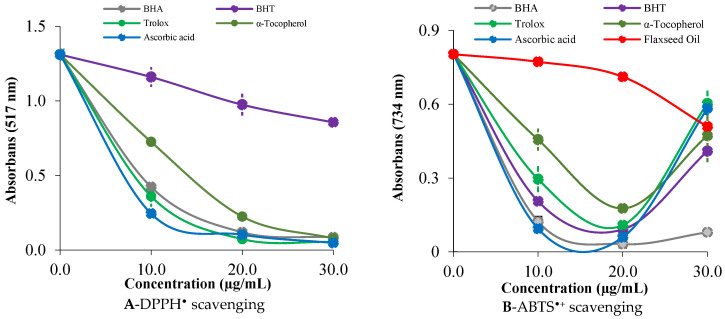
Radical scavenging potentials of steam-distilled oil of *L. usitatissimum* (SDOLU) and standards on DPPH^•^ (**A**) and ABTS^•+^ (**B**) assays.

The antioxidant capacity of bioactive substances is commonly assessed using DPPH^•^ and ABTS^•+^ scavenging assays, which provide insights into their ability to counteract oxidative stress and prevent related chronic diseases [15]. In the DPPH radical scavenging assay, SDOLU demonstrated a strong free radical scavenging effect, with an IC_50_ value of 19.80 µg/mL (r^2^ = 0.9998). Although this value is higher than those of several standard antioxidants—ascorbic acid (5.82 µg/mL, r^2^ = 0.9668), Trolox (6.03 µg/mL, r^2^ = 0.9925), BHA (6.86 µg/mL, r^2^ = 0.9949), and α-tocopherol (7.70 µg/mL, r^2^ = 0.9961)—it is significantly lower than that of BHT (49.50 µg/mL, r^2^ = 0.9957), a widely used synthetic antioxidant. These results underscore the potent radical scavenging capacity of SDOLU and support its potential as a natural antioxidant source for use in food and pharmaceutical applications (Figure 3A and Table 3).

In Figure 3B, the ABTS^•+^ radical scavenging activity of SDOLU is shown to increase in a concentration-dependent manner, with a significant rise observed between 10 and 20 µg/mL (*p* < 0.001). The IC_50_ value for SDOLU in the ABTS^•+^ assay was calculated as 57.75 µg/mL (r^2^ = 0.9887), as presented in Table 4. In comparison, standard antioxidants exhibited considerably lower IC_50_ values: BHA (6.36 µg/mL, r^2^ = 0.9746), ascorbic acid (11.75 µg/mL, r^2^ = 0.9983), BHT (12.60 µg/mL, r^2^ = 0.9995), Trolox (16.50 µg/mL, r^2^ = 0.9775), and α-tocopherol (18.73 µg/mL, r^2^ = 0.9347). Although SDOLU showed a higher IC_50_ compared to the standard antioxidants, it still demonstrated considerable ABTS^•+^ radical scavenging activity, supporting its potential as a natural antioxidant source (Figure 3B and Table 4).

### 2.4. Evaluation of Enzyme Inhibition Effects of Steam-Distilled Oil of L. usitatissimum

Table 4 presents the enzyme inhibitory activities of steam-distilled oil of SDOLU against key therapeutic targets. For acetylcholinesterase (AChE), the oil exhibited an IC_50_ value of 13.23 µM (r^2^ = 0.9839), indicating a moderate inhibitory effect. In comparison, tacrine, a standard AChE inhibitor, showed a stronger inhibition with an IC_50_ of 8.82 µM (r^2^ = 0.9836). SDOLU also demonstrated moderate inhibition against α-amylase, with an IC_50_ value of 531.44 µM (r^2^ = 0.9194), as summarized in Table 5. Additionally, SDOLU showed activity against the cytosolic and physiologically dominant isoenzyme hCA II, with an IC_50_ value of 281.02 µM (r^2^ = 0.9148). For comparison, acetazolamide (AZA), a clinically approved carbonic anhydrase inhibitor, exhibited a significantly stronger effect, with an IC_50_ of 9.96 µM (r^2^ = 0.9930). These results suggest that SDOLU possesses measurable enzyme inhibitory potential, particularly relevant to neurodegenerative and metabolic disorder targets.

## 3. Discussion

Flaxseed is a rich source of oil (32–45%), with α-linolenic acid accounting for over half of the oil content (51–55%). It also provides bioactive lignans, particularly secoisolariciresinol diglucoside (SDG), which is largely responsible for its antioxidant and metabolic benefits. Though its preventative effects on diabetes are not yet conclusive, SDG has shown strong potential in improving blood glucose regulation. In animal studies, SDG lowered the risk of type-1 diabetes (T1DM) by around 75% in both Streptozotocin (STZ)-induced and BBdp rat models, largely by reducing oxidative stress markers such as malondialdehyde (MDA). In T2DM models using ZDF rats, SDG delayed disease onset and reduced both MDA and HbA1c levels. These findings support the therapeutic potential of flaxseed-derived SDG in managing and possibly preventing diabetes [49].

Overweight and obesity are key risk factors for diseases like diabetes. Though rates have slightly dropped in some developing countries, they are expected to rise by 2030 [50,51]. Excess fat increases inflammatory adipocytes, leading to metabolic problems [52]. Small lifestyle and diet changes, especially in prediabetes, can help delay these issues [53]. Flaxseed oil, rich in ω-3 and α-linolenic acid (ALA), reduces inflammation by regulating adipokines and supports healthy fat tissue. It also has anti-inflammatory, antioxidant, and anti-atherosclerotic benefits. Unlike some studies suggesting flaxseed benefits inflammation and metabolism in obesity and diabetes, this study found no clear effect. Differences in dose, duration, and participants may explain the mixed results. Further research is needed to confirm its role [54].

Oxidative stress significantly contributes to the development and progression of T2DM [55]. Haliga et al. showed that in an STZ-induced diabetic hamster model, dietary flaxseed reduced renal oxidative stress by boosting SOD activity and lowering thiobarbituric acid reactive substances (TBARS) [56]. Similarly, this study found that flaxseed oil supplementation raised serum SOD levels and decreased MDA. These findings emphasize the connection between oxidative stress and T2DM and support the potential of a healthy diet to alleviate the disease [57].

Type-2 diabetes (T2DM) worsens cognitive decline and impairs insulin signaling in Alzheimer’s disease (AD), with both conditions involving disrupted glucose metabolism-T2DM through chronic hyperglycemia and AD through impaired neuronal glucose uptake. This overlap has led to AD being referred to as “type-3 diabetes”, highlighting altered brain glucose utilization [58,59]. Interestingly, a 12-week trial in healthy older adults showed that daily flaxseed oil (2.2 g ALA) improved verbal fluency, suggesting ALA may support executive function and cognitive health in aging. Zhu and colleagues demonstrated that dietary flaxseed oil improved STZ-NA-induced T2DM in rats by reducing inflammation, modulating gut microbiota, and boosting acetate levels, indicating its potential as an affordable strategy for diabetes prevention and treatment [60].

Consumption of ω-3 PUFAs benefits chronic metabolic diseases like T2DM by reducing inflammation and oxidative stress, as shown in clinical and experimental studies [61,62]. Flaxseed oil, a rich plant-based source of ω-3 PUFAs (especially ALA), has been widely studied for its anti-inflammatory effects throughout the body [62,63]. Low-grade inflammation characterizes diabetes, a chronic metabolic disease [64]. Cytokines like IL-1β, IL-6, and TNF-α are released by activated immune cells, impairing insulin secretion and causing metabolic dysfunction. Among these, TNF-α is known to promote insulin resistance [65]. In chronic diseases, ω-3 fatty acids are known for their anti-inflammatory properties [66]. High glucose levels in T2DM promote the formation of advanced glycation end products (AGEs), which activate NF-κB and increase TNF-α, driving chronic inflammation [67]. Supplementation with flaxseed oil has been shown to reduce glucose intolerance and lower inflammatory cytokines in diabetic models [68]. Additionally, dietary ω-3 intake decreases inflammatory markers and suppresses immune cell activity [69]. Another study explored the effects of n-3 fatty acids from flaxseed oil on genetic and metabolic parameters in women with gestational diabetes mellitus. After 6 weeks of supplementation, improvements were observed in the expression of genes involved in insulin function, lipid metabolism, glycemic regulation, inflammatory responses, and oxidative stress [70].

Natural compounds and plant-derived secondary metabolites have attracted growing interest for the treatment of T2DM due to their antioxidant, anti-inflammatory, and glucose-lowering properties [71]. These bioactive substances help regulate blood sugar levels, improve insulin sensitivity, and reduce oxidative stress [72]. Another study demonstrated that natural compounds like flavonoids, polyphenols, and ω-3 fatty acids contribute to metabolic regulation in diabetes and CVD. Their effects—such as lowering blood glucose, reducing inflammation, and improving vascular function—highlight their therapeutic potential. However, further studies are needed to confirm their clinical effectiveness [73]. Flaxseed oil has shown benefits in lipid metabolism, but its role in insulin resistance is unclear. This study found that flaxseed oil improved high-fat diet-induced hepatic steatosis, insulin resistance, and inflammation in mice. These effects were linked to enhanced n-3 fatty acid remodeling, improved insulin signaling, and restoration of ER stress and JNK pathways [74]. Flaxseed oil showed protective effects in streptozotocin-nicotinamide–induced diabetic rats by reducing renal lipid peroxidation and upregulating antioxidant enzymes (SOD-1, GPx-1, and CAT). It also downregulated inflammatory markers (IL-6, NF-κB, HO-1, and RAGE) and limited AGE formation, suggesting its potential to slow diabetic nephropathy progression [75]. Flaxseed oil improves motor function, memory, and neuronal structure disrupted by a high-ammonium diet, likely through its anti-inflammatory effects. It helps restore neuronal, cognitive, and motor function in rats with hyperammonemia [76].

Other study investigated the neuroprotective effects of flaxseed oil against cadmium-induced neurotoxicity in rats. Cadmium impaired learning, memory, and brain biochemistry, increasing MDA, NO, AChE, caspase-3, and Bcl-2 levels while reducing GSH. Flaxseed oil improved cognitive function, restored oxidative balance, lowered apoptotic markers, and protected brain tissue. These findings suggest flaxseed oil helps prevent cadmium-related neurotoxicity by enhancing antioxidant defenses and reducing neuronal death [77]. Consumption of flaxseed oil has been linked to a reduced risk of heart disease, lower plasma cholesterol and blood pressure, and improvement in central nervous system symptoms such as behavioral despair and anhedonia. Its polyunsaturated fatty acids (PUFAs), particularly α-linolenic acid, provide strong anti-inflammatory, immunoregulatory, antibacterial, and bone-strengthening effects by enhancing bone mineral density and strength. In addition to PUFAs, other components like tocopherols, β-carotene, phytosterols, and polyphenols contribute to the oil’s antioxidant, anticancer, and overall protective properties [78].

Another study demonstrated that flaxseed oil pretreatment in ovariectomized rats effectively reduced behavioral disturbances, neuronal damage, and inflammation caused by trimethyltin chloride (TMT) exposure. Flaxseed oil treatment lowered levels of pro-inflammatory cytokines and alleviated astrogliosis and microgliosis, partly through modulation of estrogen receptor signaling pathways. These results highlight the potential of flaxseed oil as a neuroprotective agent against TMT-induced hippocampal neurodegeneration and related cognitive impairments [79]. Flaxseed oil, rich in α-linolenic acid, lignans, and fiber, is considered a “superfood” with health benefits such as improving lipid profiles, lowering blood pressure and glucose, and reducing menopausal symptoms. It also has anticancer and antioxidant effects. However, antinutrients like cyanogenic glycosides may reduce nutrient absorption. Flaxseed is best consumed ground for optimal bioavailability of its active compounds [80].

In light of the information discussed and presented above, it is evident that the broad-spectrum biological activity we have demonstrated regarding flaxseed oil is of significant importance. Another important outcome revealed by our study is the confirmation of the notably positive effects of flaxseed oil on certain metabolic diseases, such as diabetes, AD, and glaucoma, which are global and widespread health concerns. The only limitation of this study is that it was not supported by in vivo experiments due to the limitations of our current laboratory facilities. Hopefully, as our laboratory capabilities improve in the future, in vivo studies will be conducted and evaluated as a separate research project. Also, more research is needed to fully understand flaxseed oil’s long-term health benefits.

## 4. Materials and Methods

### 4.1. Chemicals

Chemical reagents used in this study were obtained from various reputable suppliers to ensure high purity and consistency. Trolox (6-Hydroxy-2,5,7,8-tetramethylchroman-2-carboxylic acid), neocuprine (2,9-dimethyl-1,10-phenanthroline), α-tocopherol, ABTS (2,2′-azino-bis(3-ethylbenzthiazoline-6-sulfonic acid)), BHT (butylated hydroxytoluene), BHA (butylated hydroxyanisole), and DPPH (1,1-diphenyl-2-picrylhydrazyl) were purchased from Sigma-Aldrich Chemie GmbH (Taufkirchen, Germany). Additional phenolic and flavonoid standards, including fumaric acid, ascorbic acid, caffeic acid, chlorogenic acid, vanillic acid, naringin, rutin, *p*-coumaric acid, syringic acid, salicylic acid, rosmarinic acid, naringenin, quercetin, chrysin, luteolin, and emodin, were also sourced from Sigma-Aldrich.

Specialized compounds such as luteolin 7-glycoside, hyperoside, (+)-*trans*-Taxifolin, orientin, apigenin, hispidulin, acacetin, and hederagenin were acquired from TRC (Burlington, ON, Canada). Luteolin-7-rutinoside and verbascoside were supplied by HWI Analytik GMBH (Rülzheim, Germany) and Carbosynth (Staad, Switzerland), respectively. Hesperidin was procured from J&K Company (City of Industry, CA, USA), and myricetin from Carl Roth GmbH & Co. Penduletin (Karlsruhe, Germany), isosakuranetin, and dihydrokaempferol were obtained from Phytolab (Vestenbergsgreuth, Germany). Apigenin 7-glucoside was sourced from EDQM CS, while caffeic acid phenethyl ester (CAPE) and nepetin were purchased from the European Pharmacopoeia (Strasbourg, France) and Supelco (Bellefonte, PA, USA), respectively.

### 4.2. Extraction of Steam-Distilled Oil of L. usitatissimum

The *Linum usitatissimum* seeds were sourced locally, and oil was extracted using steam distillation. In this process, steam passes through the seeds, vaporizing volatile constituents which are then condensed into two layers: an upper oil layer containing water-insoluble compounds and a lower hydrosol layer with water-soluble components. Cohobation was used to recover remaining polar compounds. The seeds were placed on a grid above the steam inlet for about 2 h. The condensed vapor mixture separated naturally, allowing the oil—being less dense—to be collected carefully from the container.

### 4.3. Profiling of Polyphenols in Steam-Distilled Oil of L. usitatissimum by LC-HRMS

LC-HRMS analyses were conducted using a Thermo Orbitrap Q-Exactive mass spectrometer (Thermo Fisher Scientific Inc., Waltham, MA, USA) equipped with a Troyasil C18 column (150 × 3 mm, 3 µm particle size) in Istanbul, Türkiye. The mobile phase consisted of 1% formic acid in water (Phase A) and 1% formic acid in methanol (Phase B). The gradient program was set as follows: 50% A/50% B from 0 to 1 min, 100% B from 1.01 to 6 min, and 50% A/50% B from 6.01 to 10 min. The flow rate was 0.35 mL/min with the column temperature maintained at 22 °C. Ambient conditions were controlled at 22.0 ± 5.0 °C and 50 ± 15% relative humidity. Based on prior experience and literature, an acidified methanol-water gradient was chosen to optimize ionization and separation. Electrospray ionization (ESI) was selected due to its high efficiency with small to moderately polar compounds. The mass spectrometer operated in high-resolution mode, scanning ions from *m/z* 85 to 1500. Compound identification was performed by comparing retention times and HRMS data with standards of 95–99% purity (details in Section 2.1). Dihydrocapsaicin (95% purity) served as an internal standard to enhance repeatability and correct for ionization variability. Mass parameters for each analyte are listed in Table 5. This table indicates the presence of polyphenolic compounds in SDOLU, albeit in relatively low amounts, in addition to these components. Further details on the LC-HRMS method, uncertainty analysis, and phenolic confirmation are available in previous studies [81,82,83].

Specifically, 100 mg of SDOLU was dissolved in 4 mL of mobile phase B (1% formic acid in methanol) in a volumetric flask and ultrasonicated for 10 min. Next, 100 μL of dihydrocapsaicin internal standard solution (in methanol) was added, and the volume was adjusted with mobile phase B. The solution was filtered through a 0.45 μm Millipore Millex-HV filter before transferring 1 mL to an auto-sampler vial. Samples were kept at 15 °C in the auto-sampler, and 2 μL was injected per LC-HRMS run [48].

The LC-HRMS method was validated using analytical standards for the target compounds, employing either positive or negative ionization modes as detailed in Table 5. Dihydrocapsaicin was used as the internal standard throughout the validation. Key validation parameters assessed included linearity, selectivity, recovery, intermediate precision, repeatability, limit of quantification (LOQ), and limit of detection (LOD). The LOD and LOQ were calculated using the formula: LOQ or LOD = κ × SDa/b, where κ equals 3, SDa is the standard deviation of the intercept, and b is the slope of the calibration curve. For a detailed description of the validation process and uncertainty assessment, please refer to our previous publications [81,82,83,84].

### 4.4. Analysis of Steam-Distilled Oil of L. usitatissimum by GC/MS and GC-FID

SDOLU was dried over anhydrous CaCl_2_ and stored at 4 °C until GC-MS/FID analysis. The oil yield was 1.52%. GC-MS was performed using a Thermo Scientific Trace GC 1310 system coupled with a Thermo TSQ 9610 MS (Waltham, MA, USA), equipped with a DB-5 capillary column (60 m × 0.25 mm, 0.25 µm film). Helium was used as the carrier gas at 0.8 mL/min. The oven temperature was held at 80 °C for 10 min, then increased by 4 °C/min to 280 °C and held for 5 min. Injector temperature was 250 °C with a 1:20 split ratio. Mass spectra were recorded at 70 eV over *m/z* 35–650.

GC-FID analysis was performed using the same Thermo Scientific Trace GC 1310 system, with the FID detector set at 280 °C. To ensure consistency with GC-MS data, simultaneous duplicate auto-injections were conducted under identical conditions using the same column. Compound percentages were calculated from FID and total ion chromatogram (TIC) of GC-MS chromatogram [85,86,87]. Compound identification was based on comparison of mass spectra and retention times with authentic standards, the Wiley library, NIST database, and relevant literature [86,87].

### 4.5. Reducing Capacity of Steam-Distilled Oil of L. usitatissimum

The Fe^3+^ reduction potential of SDOLU was assessed using the Fe^3+^(CN−)_6_ complex reduction method, following the protocol in a previous study [88]. Different concentrations of SDOLU were mixed with 2.5 mL phosphate buffer (0.2 M, pH 6.6) and 2.5 mL of 1% K_3_Fe(CN)_6_. After vortexing and incubation at 50 °C for 25 min, 2.5 mL of 10% trichloroacetic acid was added. A 2.5 mL aliquot of the upper layer was combined with 2.5 mL distilled water and 0.5 mL of 0.1% FeCl_3_. Absorbance was measured at 700 nm to determine the reducing power of the oil.

Various concentrations of the oil (10–30 μg/mL) were prepared to assess its Cu^2+^ reducing power following a previously described method [89]. To each test tube, 0.25 mL of 10 mM CuCl_2_, 0.25 mL of ethanolic neocuprine (7.5 × 10−^3^ M), and 0.25 mL of 1.0 M ammonium acetate buffer were added. The final volume was brought to 2 mL with distilled water. After 30 min of incubation, absorbance was recorded at 450 nm.

Various concentrations of the oil were added to test tubes to assess their Fe^3+^-TPTZ complex reducing ability, following a previously described method [90]. A fresh TPTZ solution (2.25 mL, 10 mM in 40 mM HCl) was then added, followed by 2.5 mL of acetate buffer (0.3 M, pH 3.6) and 2.25 mL of FeCl_3_ solution (20 mM). The mixture was incubated at 37 °C for 25 min. Absorbance was recorded at 593 nm to determine reducing power. All experiments were carried out in triplicate, and results were averaged for reliability.

### 4.6. Free Radical Scavenging Capacity of Steam-Distilled Oil of L. usitatissimum

The radical scavenging activity of SDOLU was evaluated using the DPPH assay, based on the method of Blois [91]. A 1 mL aliquot of 0.1 mM DPPH^•^ solution in ethanol was mixed with varying concentrations of the oil (10–30 μg/mL). After incubation at room temperature for 25 min, the absorbance was measured at 517 nm to assess the scavenging effect [92].

Various concentrations of the oil (10–30 μg/mL) were added to 1 mL of ABTS^•+^ solution, with the total volume adjusted to 3 mL. The ABTS^•+^ was generated by oxidizing a 7.0 mM ABTS solution with 2.5 mM K_2_S_2_O_8_ and then diluted with 0.1 M phosphate buffer (pH 7.4) to an absorbance of 0.750 ± 0.025 at 734 nm. After incubating the mixture for 30 min, the absorbance was recorded at 734 nm to assess the radical scavenging activity of the oil [93,94]. The ability of the oil to neutralize free radicals was quantified by calculating the radical scavenging capacity (RSC) using the following equation:

RSC (%) = (1 − Ac/As) × 100

where Ac denotes the absorbance measured in the absence of the control, and As is the absorbance with the sample present. To evaluate the potency of the oil, the IC_50_ value was derived from the plotted data, indicating the concentration at which half of the free radicals are inhibited, expressed in micrograms per milliliter [95].

### 4.7. Acetylcholinesterase Inhibitory Effects of Steam-Distilled Oil of L. usitatissimum

The potential of SDOLU to inhibit acetylcholinesterase (AChE) was investigated according to a previously published protocol [96]. In this assay, mixtures were prepared by combining 1 mL of Tris-HCl buffer (1.0 M, pH 8.0) with 10 μL of the SDOLU at different concentrations and 50 μL of AChE enzyme solution. Following a 15 min incubation at room temperature (25 °C), 50 μL of 0.5 mM DTNB was added, then 50 μL of 10 mM acetylthiocholine iodide was introduced to start the enzymatic reaction. The decrease in AChE activity was quantified by measuring the absorbance at 412 nm, reflecting the inhibitory effect of the SDOLU on the enzyme [97].

### 4.8. α-Amylase Inhibition Potential of Steam-Distilled Oil of L. usitatissimum

Using the method adapted from Xiao [98], α-amylase inhibition by SDOLU was evaluated. First, 35 µL of phosphate buffer (pH 6.9), 35 µL of starch solution, and 5 µL of fig seed oil at various concentrations were combined and incubated at 37 °C for 20 min. The starch solution was prepared by dissolving 1 g of starch in 50 mL of 0.4 M NaOH, heating at 80 °C for 20 min, then cooling and adjusting the pH to 6.9 with distilled water before making the volume up to 100 mL. After the initial incubation, 20 µL of α-amylase enzyme was added to the mixture, followed by another 20 min incubation. The reaction was stopped by adding 50 µL of 0.1 M HCl, and the absorbance was measured at 580 nm to assess α-amylase inhibition [99].

### 4.9. Carbonic Anhydrase II (hCA II) Inhibition Effects of Steam-Distilled Oil of L. usitatissimum

Human carbonic anhydrase II (hCA II) was initially isolated from human erythrocytes following the procedure described by [100]. To obtain a high-purity hCA II isoenzyme, the protein underwent further purification using a Sepharose-4B-*L*-Tyrosine-Sulfanilamide affinity chromatography method [101]. Protein concentrations at each purification stage were measured by the Bradford assay [102], with bovine serum albumin as the standard [103]. The purity of the isolated hCA II isoenzyme was confirmed via SDS-PAGE according to our previous protocol [104]. Throughout the purification and inhibition assays, esterase activity was monitored spectrophotometrically at 348 nm to assess enzyme functionality [105].

### 4.10. IC_50_ Value Determination

The inhibitory potency of SDOLU was assessed by calculating the IC_50_ values. These were derived from enzyme activity measurements showing dose-dependent inhibition with increasing SDOLU concentrations [106]. IC_50_ values were determined by plotting the activity data and identifying the concentration that reduced enzyme activity by 50% [106].

### 4.11. Data Analysis and Statistics

Data were analyzed using Student’s *t*-test with GraphPad Prism 6 software (version 7.0, GraphPad, La Jolla, CA, USA). Results are expressed as mean ± standard deviation (SD), and statistical significance was determined at *p* < 0.05.

## 5. Conclusions

This study comprehensively evaluated the bioactive potential of steam-distilled oil of *L. usitatissimum* (SDOLU) through a series of in vitro assays targeting its antioxidant capacity and inhibitory effects on critical enzymes linked to AD, diabetes, and glaucoma, namely AChE, α-amylase, and hCA II. Advanced analytical techniques, including LC-HR/MS and GC/MS, were utilized to profile the oil’s chemical constituents. Key phenolic compounds such as Epigallocatechin and Naringenin were identified, alongside major fatty acid components like linolenic and linoleic acid and plant-based steroids, which comprised a significant portion of the oil’s composition. Additional bioactive molecules detected included Palmitic acid, 2,4-di-*tert*-butylphenol, Stenol, and Cetal, with LC-HR/MS further revealing notable levels of Chrysin, Hispidulin, and Rosmarinic acid. These findings suggest that the various biologically active compounds contained in the steam-distilled oil of *L. usitatissimum* may be a powerful natural antioxidant source and may have promising effects on disorders that may occur due to oxidative stress and some metabolic diseases.

## Figures and Tables

**Table 1:** Compounds in steam-distilled oil of *L. usitatissimum* (SDOLU) identified by GC-MS.

Compounds	Formula	RT	Area (%)	Identification
*α*-Pinene	C_10_H_16_	3.36	0.53	RT, ST, MS
Cymene	C_10_H_14_	4.88	t	RT, ST, MS
1-Dodecanol	C_12_H_26_O	7.92	0.25	RT, MS
1-Tetradecene	C_14_H_28_	12.13	0.54	RT, MS
2,4,-Di-*t*-buthylphenol	C_14_H_22_O	14.63	1.24	RT, MS
Hexaadecanol (cetal)	C_16_H_34_O	16.15	0.83	RT, MS
Nonadecene	C_19_H_38_	19.83	0.94	RT, MS
Palmitic acid	C_16_H_32_O_2_	22.64	4.91	RT, MS, ST
Linoleic acid	C_18_H_32_O_2_	25.36	13.21	RT, MS, ST
Linolenic acid	C_18_H_30_O_2_	25.46	57.97	RT, MS, ST
9-Octadecenoic acid	C_18_H_34_O_2_	26.21	t	RT, MS
Stearic acid	C_18_H_36_O_2_	26.23	0.44	RT, MS, ST
Octacosanol	C_28_H_58_O	29.04	0.27	RT, MS
Campesterol	C_28_H_48_O	39.19	2.49	RT, MS
Stigmasterol	C_29_H_48_O	39.59	0.4	RT, MS
Sitosterol	C_29_H_50_O	40.40	5.2	RT, MS
Cycloartenol	C_30_H_50_O	41.55	6.56	RT, MS
	**TOTAL**		**95.78**	

RT: retention time; MS: mass spectrometry data; ST: standard compound comparison (While Ultra Kit WRK 105 terpene mixture was used for *α*-pinene and cymene, 98 > pure compounds were used for fatty acids); % is relative area ratio of total ion chromatogram (TIC), t < 0.1.

**Table 2:** The reducing power of steam-distilled oil of *L. usitatissimum* (SDOLU) at the same concentration was evaluated using multiple methods including the FRAP assay to determine ferric ion (Fe^3+^) reducing ability, and the CUPRAC assay to assess the cupric ion (Cu^2+^) reducing capacity.

Essential Oils	Fe^3+^ Reducing		Cu^2+^ Reducing		Fe^3+^-TPTZ Reducing	
	λ_700_ *	r^2^	λ_450_ *	r^2^	λ _593_ *	r^2^
BHA	2.292 ± 0.012	0.9993	2.418 ± 0.018	0.9887	1.172 ± 0.014	0.9605
BHT	2.136 ± 0.090	0.9957	1.953 ± 0.045	0.9998	0.690 ± 0.008	0.9645
Trolox	1.514 ± 0.066	0.9963	1.800 ± 0.096	0.9974	1.180 ± 0.032	0.9732
α-Tocopherol	0.862 ± 0.038	0.9996	0.851 ± 0.046	0.9994	0.918 ± 0.011	0.9904
Ascorbic acid	2.298 ± 0.086	0.9659	0.983 ± 0.048	0.9822	1.257 ± 0.024	0.9869
SDOLU	1.005 ± 0.043	0.9997	0.875 ± 0.028	0.9907	0.796 ± 0.010	0.9821

* Data are reported as the average ± standard deviation (SD) based on three independent replicates (n: 3). SDOLU: Steam-distilled oil of *L. usitatissimum.*

**Table 3:** IC_50_ values (µg/mL) for free radical scavenging activity of steam-distilled oil of *L. usitatissimum* (SDOLU) and standard antioxidants determined by ABTS^•+^ and DPPH^•^ scavenging assays.

Essential Oils	DPPH Scavenging		ABTS ^+^ Scavenging	
	IC_50_ (µg/mL)	r^2^	IC_50_ (µg/mL)	r^2^
BHA	6.86	0.9949	6.36	0.9746
BHT	49.50	0.9957	12.60	0.9995
Trolox	6.03	0.9925	16.50	0.9775
α-Tocopherol	7.70	0.9961	18.73	0.9347
Ascorbic acid	5.82	0.9668	11.75	0.9983
SDOLU	19.80	0.9998	57.75	0.9887

**Table 4:** The enzyme inhibition profile of steam-distilled oil of *L. usitatissimum* (SDOLU) and standard enzyme inhibitors.

Enzymes	SDOLU		Standards	
	IC_50_	r^2^	IC_50_	r^2^
CA II *	281.02	0.9148	9.96	0.9930
AChE *	13.23	0.9839	8.82	0.9836
α-Amylase *	531.44	0.9194	7.54	0.9074

* Acetazolamide (AZA) was utilized as the benchmark inhibitor for the carbonic anhydrase II (hCA II) isoenzyme. Tacrine was employed as the positive control in acetylcholinesterase (AChE) inhibition assays, whereas Acarbose served as the standard reference inhibitor for α-amylase activity.

**Table 5:** The chemical composition (mg/L oil) and validation parameters of steam-distilled oil of *L. usitatissimum* (SDOLU) were determined using LC-HRMS.

Phenolics	Molecular Formula	*m/z*	Ionization Mode	Linear Range	Linear Regression Equation	LOD/LOQ	R^2^	Recovery	Phenolics	U%
Epigallocatechin	C_15_H_14_O_7_	307.0812	Positive	0.3–5	y = 0.00317x + 0.000443	0.17/0.57	0.9947	102.22	1.94	3.09
Chlorogenic acid	C_16_H_18_O_9_	353.0878	Negative	0.05–10	y = 0.00817x + 0.000163	0.02/0.06	0.9994	96.68	0.42	3.58
Fumaric acid	C_4_H_4_O_4_	115.0037	Negative	0.1–10	y = 0.00061x − 0.0000329	0.05/0.17	0.9991	97.13	-	2.88
Orientin	C_21_H_20_O_11_	447.0933	Negative	0.1–10	y = 0.00757x + 0.000347	0.01/0.03	0.9993	96.22	0.26	3.67
Caffeic acid	C_9_H_8_O_4_	179.0350	Negative	0.3–10	y = 0.0304x + 0.00366	0.08/0.27	0.9993	94.51	-	3.74
Luteolin 7-glycoside	C_21_H_20_O_11_	447.0933	Negative	0.1–7	y = 0.0162x + 0.00226	0.01/0.03	0.9961	96.31	0.17	4.14
Rutin	C_27_H_30_O_16_	609.1461	Negative	0.05–10	y = 0.00329x − 0.00005576	0.01/0.03	0.999	96.97	0.19	3.07
Hyperoside	C_21_H_20_O_12_	463.0882	Negative	0.05–10	y = 0.0072x − 0.00003096	0.01/0.03	0.9995	96.62	0.28	3.46
Apigenin 7-glycoside	C_21_H_20_O_10_	431.0984	Negative	0.3–7	y = 0.0246x + 0.00306	0.01/0.03	0.9962	96.07	0.08	3.59
Ellagic acid	C_14_H_6_O_8_	300.9990	Negative	0.05–10	y = 0.0085x − 0.000612	0.03/1	0.9994	101.49	-	4.20
Quercitrin	C_21_H_20_O_11_	447.0933	Negative	0.05–10	y = 0.0179 + 0.0003331	0.01/0.03	0.999	97.00	0.12	3.78
Quercetin	C_15_H_10_O_7_	301.0354	Negative	0.1–10	y = 0.0509x + 0.00467	0.01/0.03	0.9978	96.41	-	2.95
Herniarin	C_10_H_8_O_3_	177.0546	Positive	0.1–7	y = 0.309x + 0.0266	0.01/0.03	0.9983	92.92	-	3.89
Naringenin	C_15_H_12_O_5_	271.0612	Negative	0.1–10	y = 0.0281x + 0.00182	0.01/0.03	0.9995	86.65	1.22	4.20
Luteolin	C_15_H_10_O_6_	285.0405	Negative	0.1–10	y = 0.117x + 0.00848	0.01/0.03	0.9981	96.98	0.11	3.42
Apigenin	C_15_H_10_O_5_	269.0456	Negative	0.3–10	y = 0.104x + 0.0199	0.01/0.03	0.9998	81.55	0.20	2.87
Hispidulin	C_16_H_12_O_6_	301.0707	Positive	0.05–10	y = 0.02614x + 0.0003114	0.01/0.03	0.9993	98.36	0.72	3.41
Penduletin	C_18_H_16_O_7_	343.0823	Negative	0.3–10	y = 0.0258x + 0.00253	0.01/0.03	0.9991	83.43	0.03	3.20
CAPE	C_17_H_16_O_4_	283.0976	Negative	0.3–7	y = 0.255x + 0.0477	0.01/0.03	0.9964	94.42	-	3.13

## Data Availability

Data available in a publicly accessible repository.
